# Sex-Dependent Motor Deficit and Increased Anxiety-Like States in Mice Lacking Autism-Associated Gene *Slit3*

**DOI:** 10.3389/fnbeh.2018.00261

**Published:** 2018-11-13

**Authors:** Su Mi Park, Céline Plachez, Shiyong Huang

**Affiliations:** ^1^Laboratory of Neural Circuits & Behavior, Program in Neuroscience, Hussman Institute for Autism, Baltimore, MD, United States; ^2^Autism & Brain Development Laboratory, Program in Neuroscience, Hussman Institute for Autism, Baltimore, MD, United States

**Keywords:** autism(ASD), *Slit3*, anxiety, hypolocomotion, motor coordination

## Abstract

Altered neuronal connectivity has been implicated in the pathophysiology of Autism Spectrum Disorder (ASD). SLIT/ROBO signaling plays an important role in developmental processes of neuronal connectivity, including axon guidance, neuronal migration, and axonal and dendritic branching. Genetic evidence supports that *SLIT3*, one of the genes encoding SLITs, is associated with ASD. Yet the causal link between *SLIT3* mutation and autism symptoms has not been examined. Here we assessed ASD-associated behaviors in *Slit3* knockout (KO) mice. Our data showed that *Slit3*-KO mice exhibited reduced marble burying behaviors but normal social behaviors. In addition, *Slit3*-KO mice displayed hypolocomotion in the open field test and impaired motor coordination in the rotarod test. Anxiety-like behaviors were mainly observed in female KO mice assessed by three types of behavioral tests, namely, the open field test, elevated plus maze test, and light/dark box test. No differences were observed between KO and wildtype mice in recognition memory in the novel object recognition test or depression-like behavior in the tail suspension test. Taken together, loss of *Slit3* may result in disrupted neural circuits related to motor function and increased anxiety-like states, which are co-occurring symptoms in ASD.

## 1. Introduction

SLITs, encoded by the genes *Slit1-3*, are chemorepellents that bind to Roundabout (ROBO) receptors to guide axons to find correct pathways during brain development (Brose et al., 1999; Nguyen-Ba-Charvet and Chédotal, 2002; Long et al., 2004). SLIT/ROBO signaling plays an important role in neuronal migration, cell proliferation, and axonal and dendritic branching, which are required for the normal development of neuronal connectivity (Hu, 1999; Wang et al., 1999; Shu and Richards, 2001; Whitford et al., 2002; Miyashita et al., 2004; Plachez and Richards, 2005; Andrews et al., 2007; Plachez et al., 2008; Borrell et al., 2012). For instance, the formation of thalamocortical, corticofugal, and callosal connections partially or completely depends on SLIT family (Bagri et al., 2002; Unni et al., 2012) and ROBO1/2 receptors (Lopez-Bendito et al., 2007). Altered neuronal connectivity is a potential neural signature of Autism Spectrum Disorder (ASD) (Menon, 2013; Mohammad-Rezazadeh et al., 2016). Studies using functional magnetic resonance imaging (fMRI), electroencephalography (EEG), or magnetoencephalography (MEG) found hyperconnectivity and hypoconnectivity in brains of ASD patients (Lynch et al., 2013; Di Martino et al., 2014; O'Reilly et al., 2017). Along with altered functional connectivity, brain structural abnormalities were also discovered in ASD (Freitag et al., 2009; Hardan et al., 2009; Oblak et al., 2011; Uddin et al., 2011). These lines of evidence suggest that functional changes in the SLIT/ROBO pathway may hinder the normal development of neural connectivity, resulting in neurodevelopmental conditions such as ASD.

Indeed, aberrant SLIT/ROBO signaling has been implicated in the pathogenesis of ASD (Anitha et al., 2008; Blockus and Chédotal, 2014; Perez et al., 2016). It has been shown that mRNA and protein expression of *ROBO2*, is reduced in the anterior cingulate cortex of ASD postmortem brains (Suda et al., 2011). In addition, *ROBO1* gene expression is downregulated in lymphoblastoid cell lines derived from children with autism (Hu et al., 2006). Gene variations of *ROBO3/4* and increased expression of *SLIT1* are also associated with autism (Anitha et al., 2008; Bakos et al., 2015).

Importantly, *SLIT3* is suggested as an autism-risk gene by several genetic studies. The primary evidence is from a whole- exome sequencing study in which single nucleotide variance of *SLIT3* was identified in two multiplex ASD families (Cukier et al., 2014). Single nucleotide variance of *SLIT3* was also reported in the simplex ASD family from analyses using the Simons Simplex Collection data set (Krupp et al., 2017). Additionally, the chromosomal abnormality of 5q35, where *SLIT3* is located, is linked to neurodevelopmental disorders (Schafer et al., 2001; Schiffer et al., 2003; Bækvad-Hansen et al., 2006; Menten et al., 2006). In line with this notion, it has been reported that *SLIT3* mutations are associated with schizophrenia (Shi et al., 2004) and major depressive disorder (Glessner et al., 2010), which may be attributed to the overlapped genetic susceptibility between ASD and other neuropsychiatric disorders (Carroll and Owen, 2009; Cukier et al., 2014; McCarthy et al., 2014; Khanzada et al., 2017). However, despite these indications, no study to date has examined whether mutations of *SLIT3* leads to ASD-related symptoms.

In this study, several ASD-associated behaviors in *Slit3*-KO mice were examined, including motor behaviors (open field test and rotarod test), anxiety-like behaviors (light/dark box and elevated plus maze test), depression-like behaviors (tail suspension test), cognitive ability (novel object recognition test), social behaviors (three chamber test), and repetitive behaviors (marble burying test). Behavioral assessments in *Slit3*-KO mice revealed that the absence of *Slit3* resulted in increased anxiety-like behaviors and altered motor behaviors. Since behavioral criteria are relevant to the diagnosis of ASD, *Slit3*-KO mice with behavioral characterization may be a useful animal model for the study of clinical interventions in autism.

## 2. Results

### 2.1. Normal social approach and preference behaviors in *Slit3*-KO mice

**Figure 1:**
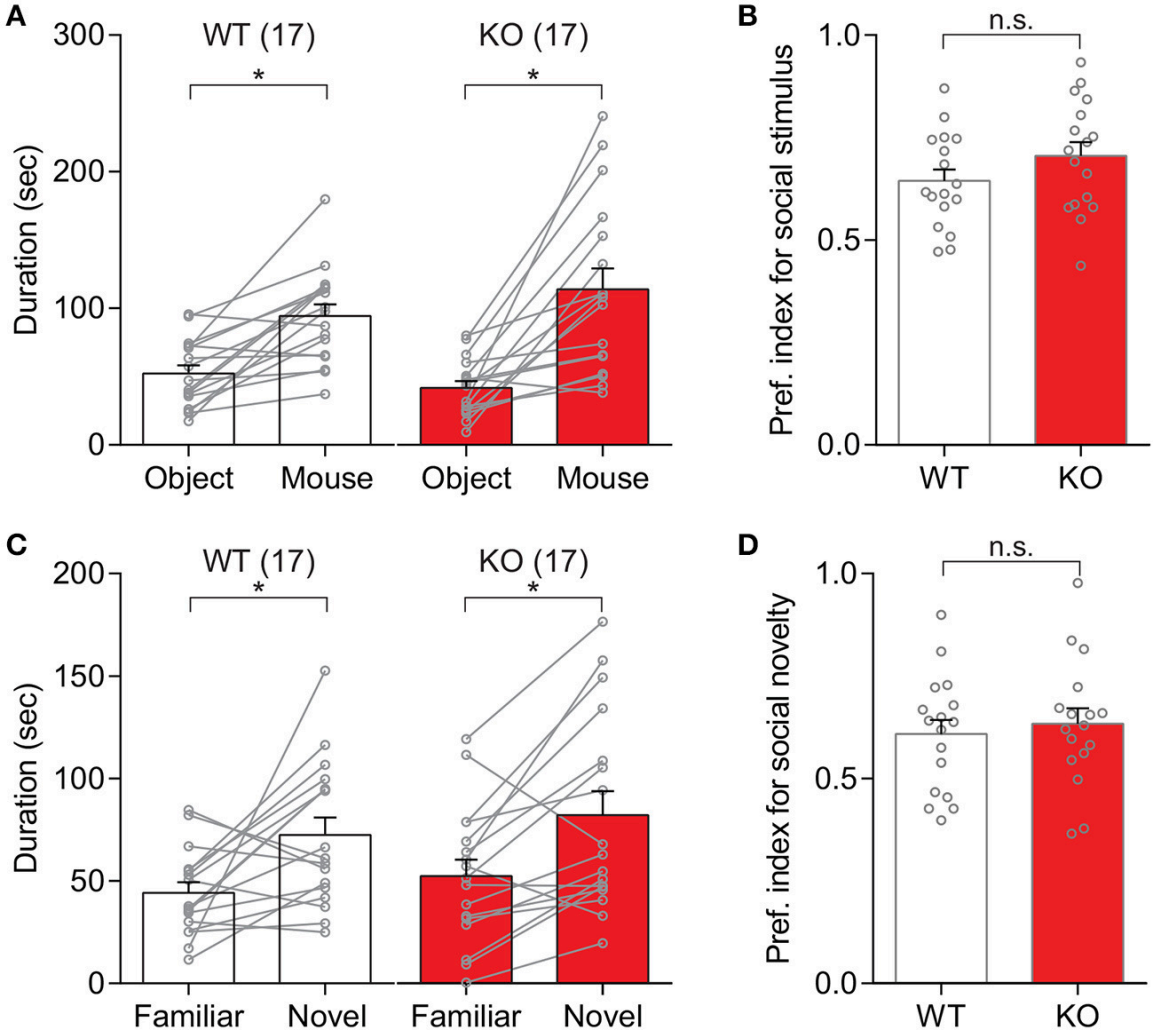
*Slit3*-KO mice showed normal social behavior in the three-chamber social test. **(A)** Both WT and KO mice spent longer time around the cup with a novel mouse than the cup with an object. WT: *p < 0.001, KO: *p < 0.001, *t*-test. **(B)** No significant difference in preference index for social stimulus between WT and KO mice. n.s., *p* = 0.167, *t*-test. **(C)** Mice spent longer time around the cup with a novel mouse than with a familiar mouse regardless of their genotypes. WT: *p = 0.012, KO: *p = 0.003, *t*-test. **(D)** No significant difference in preference index for social novelty between WT and KO mice. n.s.: *p* = 0.619, *t*-test. The preference index was calculated as Equation 1. The data in the bar chart are presented as mean ± SEM. Sample size is indicated in parentheses.

In order to examine whether the deletion of *Slit3* results in any deficit of social behaviors, social approach and preference behaviors in *Slit3*-KO mice were assessed using the three-chamber social test. As shown in Figure 1, *Slit3*-KO mice spent more time around a cup with a novel mouse than an object displayed [*t*_(16)_ = 4.84, *p* < 0.001, *t*-test], which was comparable to the WT mice [*t*_(16)_ = 4.86, *p* < 0.001, *t*-test; Figure 1A]. The preference index to social stimulus was not significantly different between WT and *Slit3*-KO mice [*t*_(32)_ = 1.42, *p* = 0.167, *t*-test; Figure 1B], suggesting normal social approach behavior in *Slit3*-KO mice. Similarly, when each sex group was analyzed separately, there was no significant genotype effect in the preference index for social stimulus [Female: *t*_(16)_ = 1.58, *p* = 0.134; Male: *t*_(14)_ = 0.40, *p* = 0.693; *t*-test]. They also exhibited normal social preference behavior as they spent more time around a cup with a novel mouse than with a familiar mouse [*t*_(16)_ = 3.51, *p* = 0.003, *t*-test], which was comparable to WT controls [*t*_(16)_ = 2.85, *p* = 0.012; Figure 1C], without any significant difference in preference to social novelty [*t*_(32)_ = 0.50, *p* = 0.619, *t*-test; Figure 1D]. Similarly, when each sex was analyzed separately, there was no significant genotype effect in the preference index for social novelty [Female: *t*_(16)_ = 0.19, *p* = 0.428; Male: *t*_(14)_ = 0.75, *p* = 0.620; *t*-test].

### 2.2. Suppressed repetitive behavior, hypolocomotion, and impaired motor coordination in *Slit3*-KO mice

**Figure 2:**
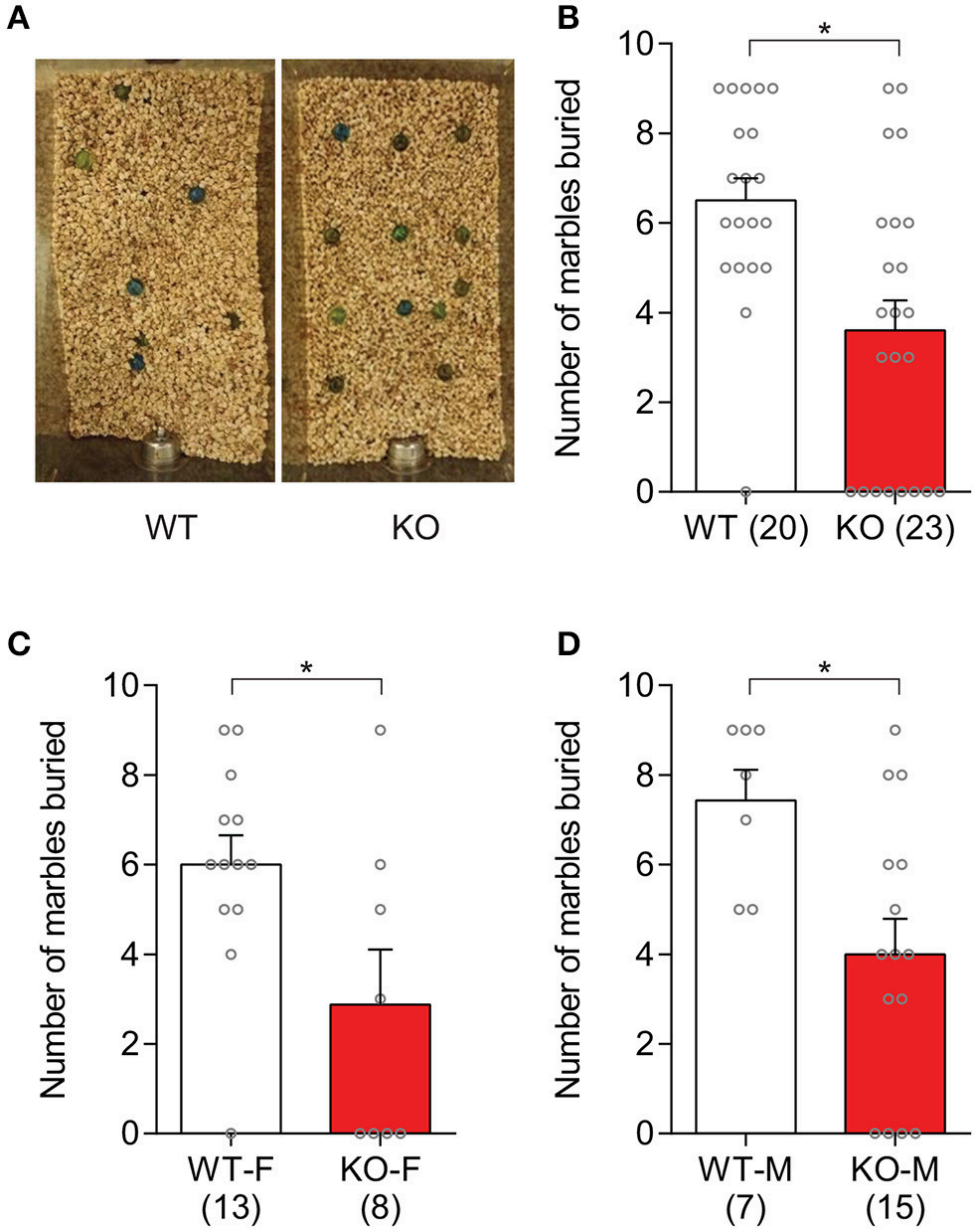
*Slit3*-KO mice showed suppressed marble burying behavior. **(A)** Example image of marbles buried by WT and KO mice. **(B)** KO mice buried significantly fewer marbles compared to WT mice. *p = 0.002, Mann–Whitney test. **(C)** Number of marbles that female mice buried in each genotype. *p = 0.041, Mann–Whitney test. **(D)** Number of marbles that male mice buried in each genotype. *p = 0.011, Mann–Whitney test. The data in the bar chart are presented as mean ± SEM. Sample size is indicated in parentheses. F, female; M, male.

Repetitive behaviors in *Slit3*-KO mice were examined by assessing animals' marble burying behavior. Our results showed that *Slit3*-KO mice buried significantly fewer marbles than WT mice (*U* = 106.5, *p* = 0.002, Mann–Whitney test; Figures 2A,B). When each sex group was analyzed separately, both female and male *Slit3*-KO mice buried fewer marbles than the WT controls (Female: *U* = 24, *p* = 0.041, Mann–Whitney test; Male: *U* = 17.5, *p* = 0.011, Mann–Whitney test; Figures 2C,D).

**Figure 3:**
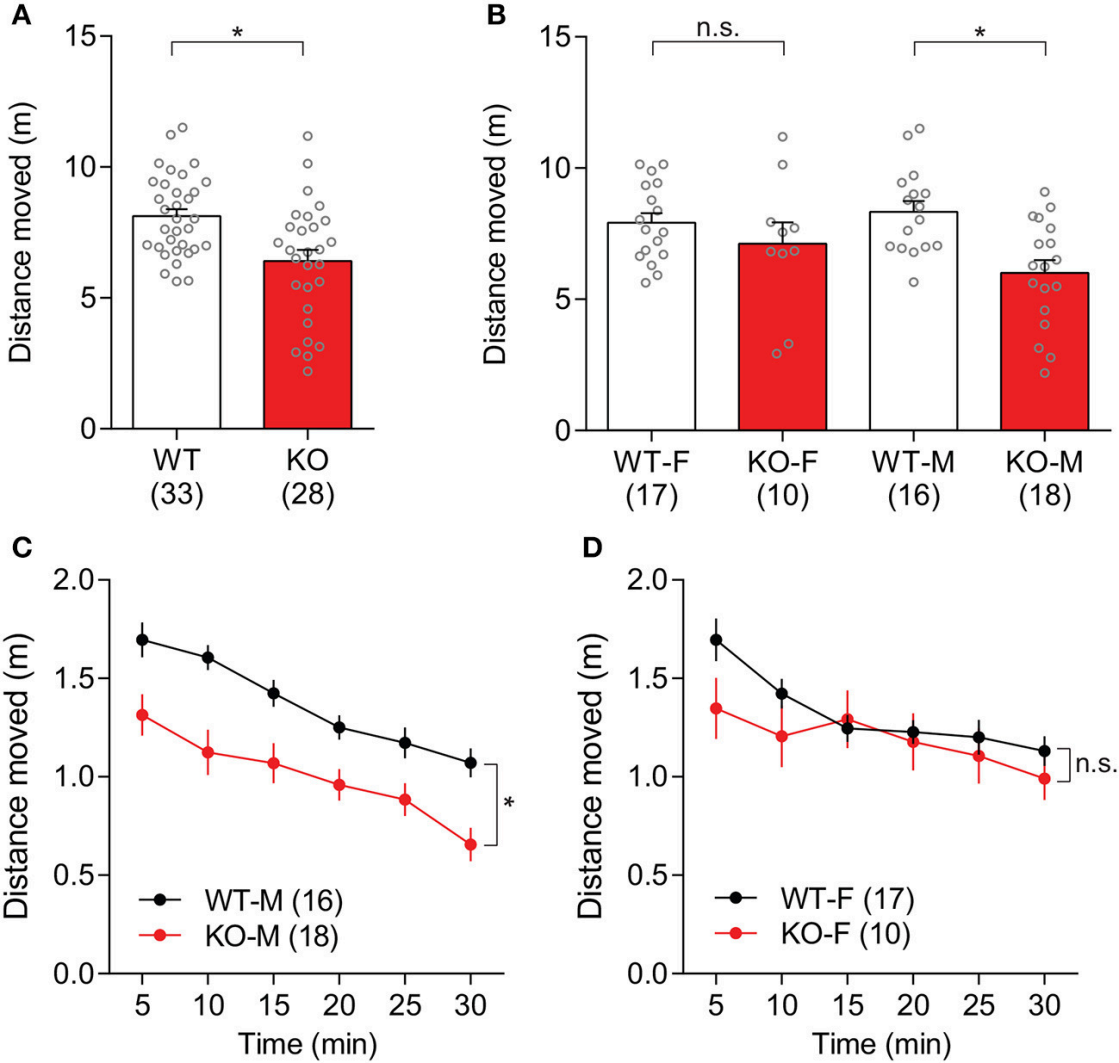
*Slit3*-KO mice were hypolocomotive in the open field test. **(A)** KO mice traveled a shorter distance than WT mice during a 30-min open field test. *p < 0.001, *t*-test. **(B)** When each sex group was analyzed separately, only male KO mice showed hypolocomotion compared to male WT mice. Male: *p < 0.001, Female: n.s.: *p* = 0.312, *t*-test. **(C)** Distance that male WT and KO mice traveled in 5-min time bin. *p = 0.001, genotype effect, two-way rmANOVA. **(D)** Distance that female WT and KO mice traveled in 5-min time bin. n.s.: *p* = 0.312, genotype effect, two-way rmANOVA. The data in the bar chart are presented as mean ± SEM. Sample size is indicated in parentheses. F, female; M, male.

General locomotive behaviors in *Slit3*-KO mice were assessed using 30-min open field tests. *Slit3*-KO mice traveled significantly shorter distances than WT mice [*t*_(59)_ = 3.50, *p* < 0.001, *t*-test; Figure 3A]. When each sex group was analyzed separately, the difference was mainly driven by male KO mice [Male: *t*_(32)_ = 3.64, *p* < 0.001, *t*-test; Female: *t*_(25)_ = 1.03, *p* = 0.312, *t*-test; Figure 3B]. Similarly, when the travel distance was binned every 5 min, male KO mice consistently traveled less distance during the 30-min testing period [*F*_(1,32)_ = 13, *p* = 0.001, genotype effect, two-way rmANOVA; Figure 3C]. The male WT and male KO mice traveled less distance as time passed, illustrating similar levels of habituation to the novel environment [*F*_(5,160)_ = 37.6, *p* < 0.001, time effect, two-way rmANOVA; Figure 3C]. In female mice, KO mice tended to travel less distance than WT mice during the first 5 min of the test, but it was not statistically significant [*t*_(25)_ = 1.88, *p* = 0.072, *t*-test; Figure 3D].

**Figure 4:**
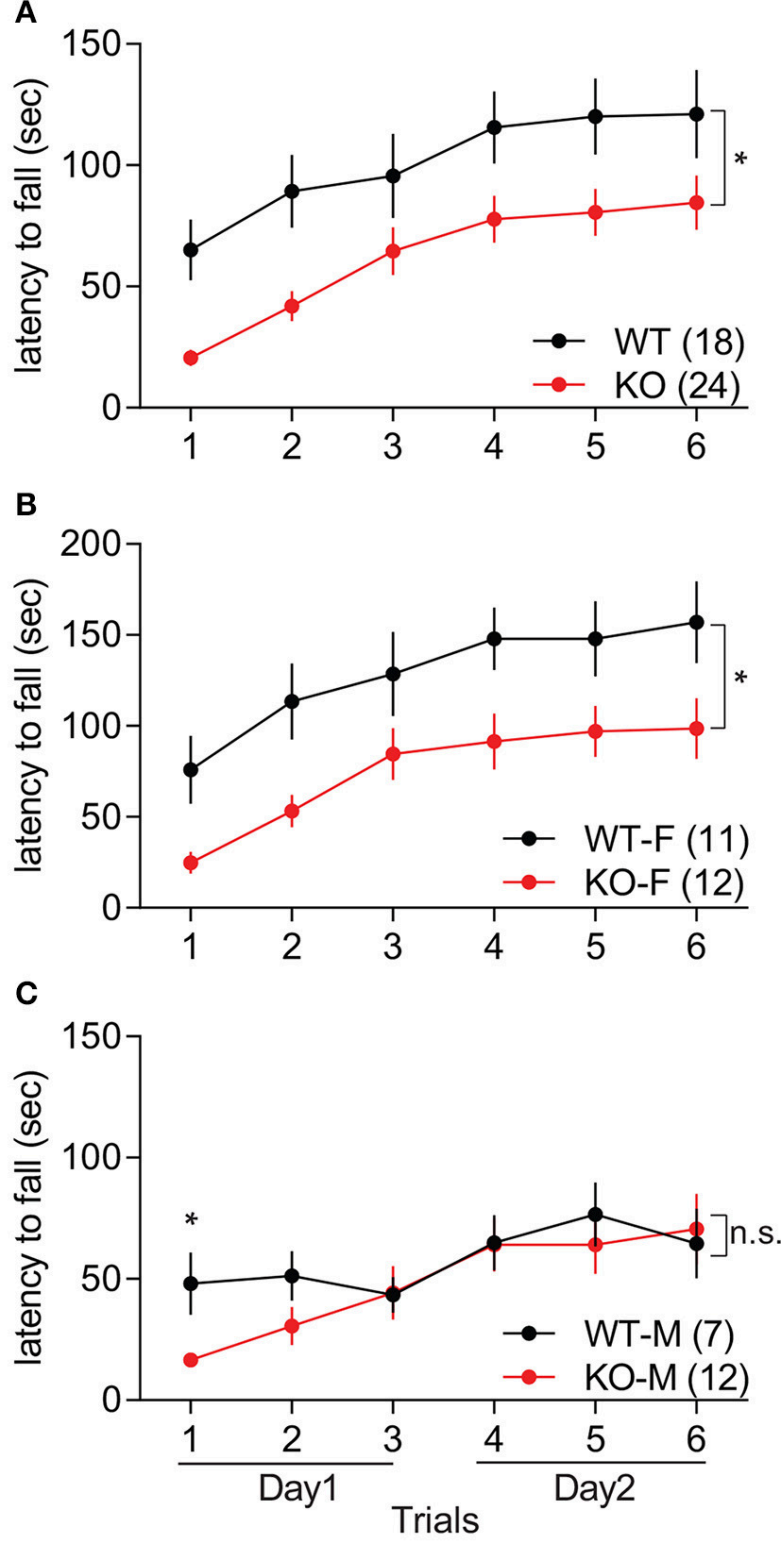
*Slit3*-KO mice displayed impaired motor coordination in the accelerating rotarod test. **(A)** KO mice showed shorter latency to fall off the rotarod than WT mice. *p = 0.014, genotype effect, two-way rmANOVA. **(B)** Latency to fall off the rotarod in female mice. *p = 0.023, genotype effect, two-way rmANOVA. **(C)** Latency to fall off the rotarod in male mice. n.s.: *p* = 0.477, genotype effect, two-way rmANOVA. *p = 0.008, *t*-test at the 1st trial. The data are presented as mean ± SEM. Sample size is indicated in parentheses. F, female; M, male.

Motor coordination in *Slit3*-KO mice was assessed during three trials of the accelerating rotarod test for 2 consecutive days. *Slit3*-KO mice showed impaired rotarod performance, as the latency to fall off the rod was shorter than WT mice over six trials in the accelerating rotarod test, indicating impaired motor coordination in *Slit3*-KO mice [*F*_(1, 40)_ = 6.63, *p* = 0.014, genotype effect, two-way rmANOVA; Figure 4A]. However, as trials were repeated, both the WT and KO mice remained on the rod for longer periods of time, suggesting normal learning rate in the KO mice [*F*_(5, 200)_ = 35.4, *p* < 0.001, trial effect, two-way rmANOVA; Figure 4A]. When each sex group was analyzed separately, only female KO mice showed impaired rotarod performance compared to female WT mice [*F*_(1, 21)_ = 5.99, *p* = 0.023, genotype effect, two-way rmANOVA; Figure 4B], while the performance of the male KO mice was not significantly different from WT controls [*F*_(1, 17)_ = 0.53, *p* = 0.477, genotype effect, two-way rmANOVA; Figure 4C]. However, both male and female mice displayed longer latency to fall off the rod in the later trials regardless of the genotype, indicating normal learning rate in the KO mice [Male: *F*_(5, 85)_ = 9.30, *p* < 0.001; Female: *F*_(5, 105)_ = 29.5, *p* < 0.001; trial effect, two-way rmANOVA; Figures 4B,C]. Although the performance of male KO mice over the trials was similar to WT mice in the rmANOVA analysis, the latency to fall off the rod in the first trial was significantly shorter than controls, suggesting impaired motor coordination in the KO mice [*t*_(17)_ = 3.01, *p* = 0.008, *t*-test; Figure 4C].

### 2.3. Increased anxiety-like behaviors in *Slit3*-KO mice

**Figure 5:**
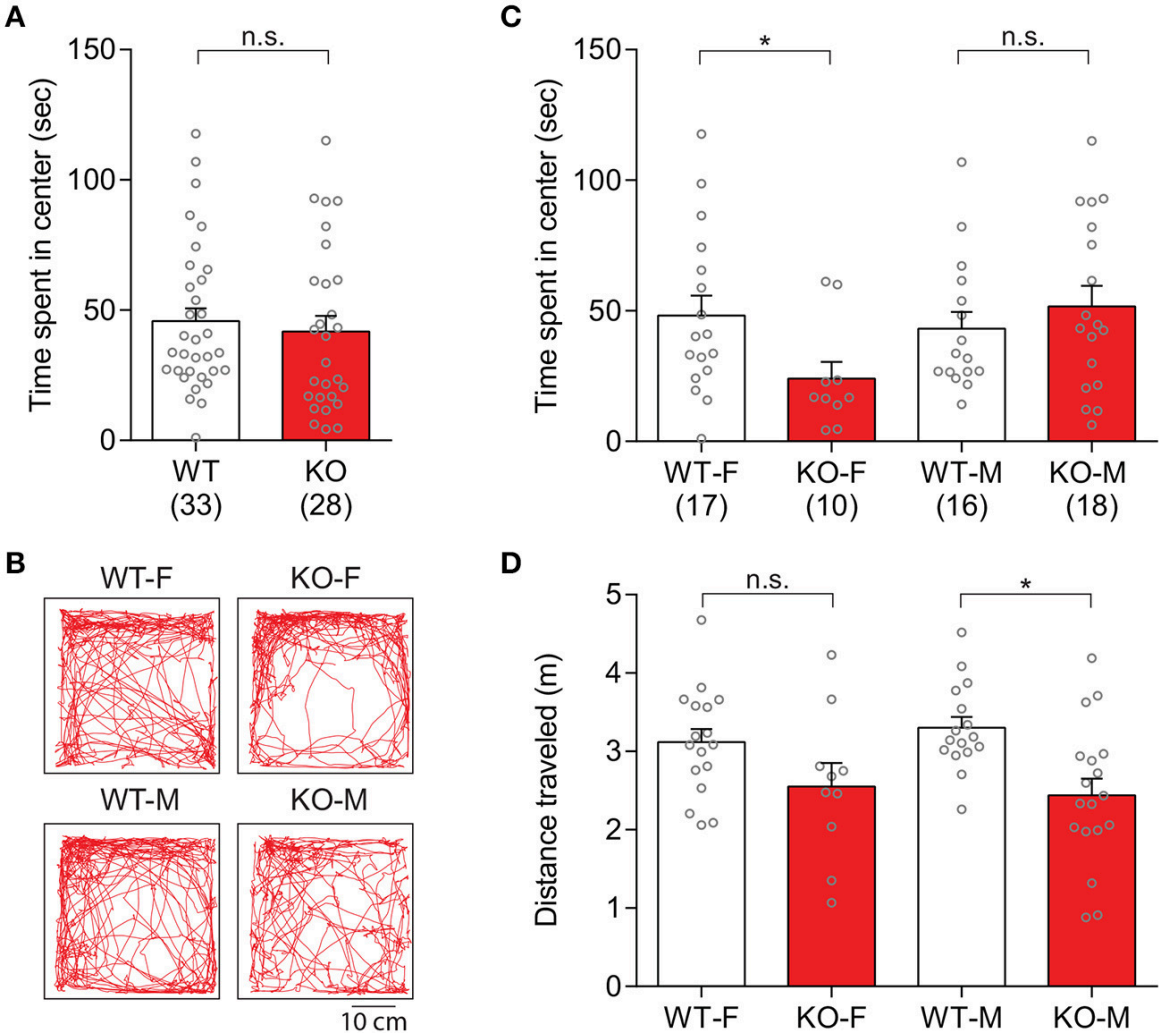
*Slit3*-KO mice displayed anxiety-like behaviors during the first 10 mins of the open field test. **(A)** Duration that each genotype group spent in the center arena of the open field. n.s.: *p* = 0.614, *t*-test. **(B)** Representative maps illustrated mice's track in the open field during the first 10 min of the test. **(C)** Female KO mice spent less time in the center arena of the open field compared to female WT mice. Female: *p = 0.041, Male: n.s.: *p* = 0.409, *t*-test. **(D)** Distance that animals traveled during the first 10 min of the test. Female: n.s.: *p* = 0.085, Male: *p = 0.002, *t*-test. The data in the bar chart are presented as mean ± SEM. Sample size is indicated in parentheses. F, female; M, male.

In order to assess anxiety-like behaviors in *Slit3*-KO mice, explorative behavior in the center of the arena during the first 10 min of the open field test was analyzed. There was no significant genotype difference in the duration of time that animals spent in the center arena [*t*_(59)_ = 0.51, *p* = 0.614, *t*-test; Figure 5A]. When each sex group was analyzed separately, female KO mice spent significantly less time in the center arena than female WT mice, indicating increased anxiety-like states in the female KO mice [*t*_(25)_ = 2.16, *p* = 0.041, *t*-test; Figures 5B,C]. The duration that male KO mice spent in the center arena was not significantly different from the male WT mice [*t*_(32)_ = 0.84, *p* = 0.409, *t*-test; Figures 5B,C]. The distance that the animals traveled during the first 10 min of the test within each sex group was also analyzed to examine the influence of possible hypolocomotion on the strong thigmotaxis shown only in female KO mice. The distance that female KO mice traveled was not significantly different from female WT mice, although there was a tendency of hypolocomotion [*t*_(25)_ = 1.79, *p* = 0.085, *t*-test; Figure 5D], suggesting a heightened anxiety-like state but a normal activity level in female *Slit3*-KO mice.

**Figure 6:**
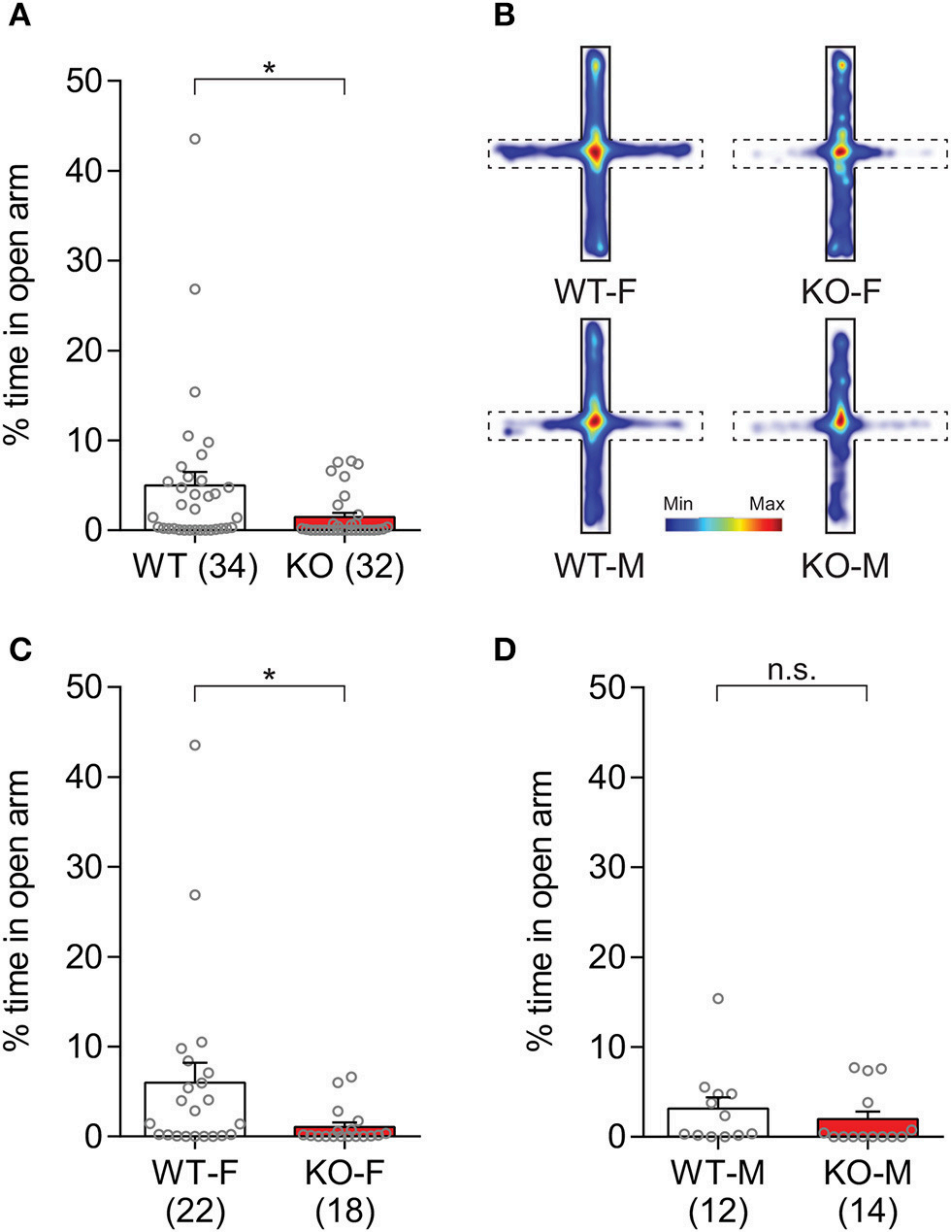
*Slit3*-KO mice displayed anxiety-like traits in the elevated plus maze test. **(A)** KO mice spent less percentage of time in the open arm of the maze compared to WT controls. *p = 0.007, Mann–Whitney test. **(B)** Heat map illustrating cumulative duration that all mice in each group spent in the elevated plus maze. Open arms are denoted as dash lines, and closed arms are denoted as solid lines. **(C)** Female WT mice spent more percentage of time in the open arm compared to female KO mice. *p = 0.023, Mann–Whitney test. **(D)** The percentage of time spent in the open arm was not significantly different between KO and WT male mice. n.s.: *p* = 0.233, Mann–Whitney test. The data in the bar chart are presented as mean ± SEM. Sample size is indicated in parentheses. F, female; M, male.

Anxiety-like behaviors in *Slit3*-KO mice were further assessed by measuring the duration that animals spent in the open arm of the elevated plus maze (Silverman et al., 2010). The *Slit3*-KO mice spent reduced percentage of time in the open arm of the maze compared to WT mice (*U* = 338, *p* = 0.007, Mann–Whitney test; Figure 6A), indicating heightened anxiety-like states in the KO mice. When each sex group was analyzed separately, female KO mice spent less percentage of time in the open arm of the maze than female WT mice, while male KO mice spent comparable time in the open arm to the controls (Female: *U* = 115, *p* = 0.023; Male: *U* = 61, *p* = 0.233; Mann–Whitney test; Figures 6B–D), suggesting increased anxiety-like states only in the female KO mice as seen in the open field tests (Figure 5).

**Figure 7:**
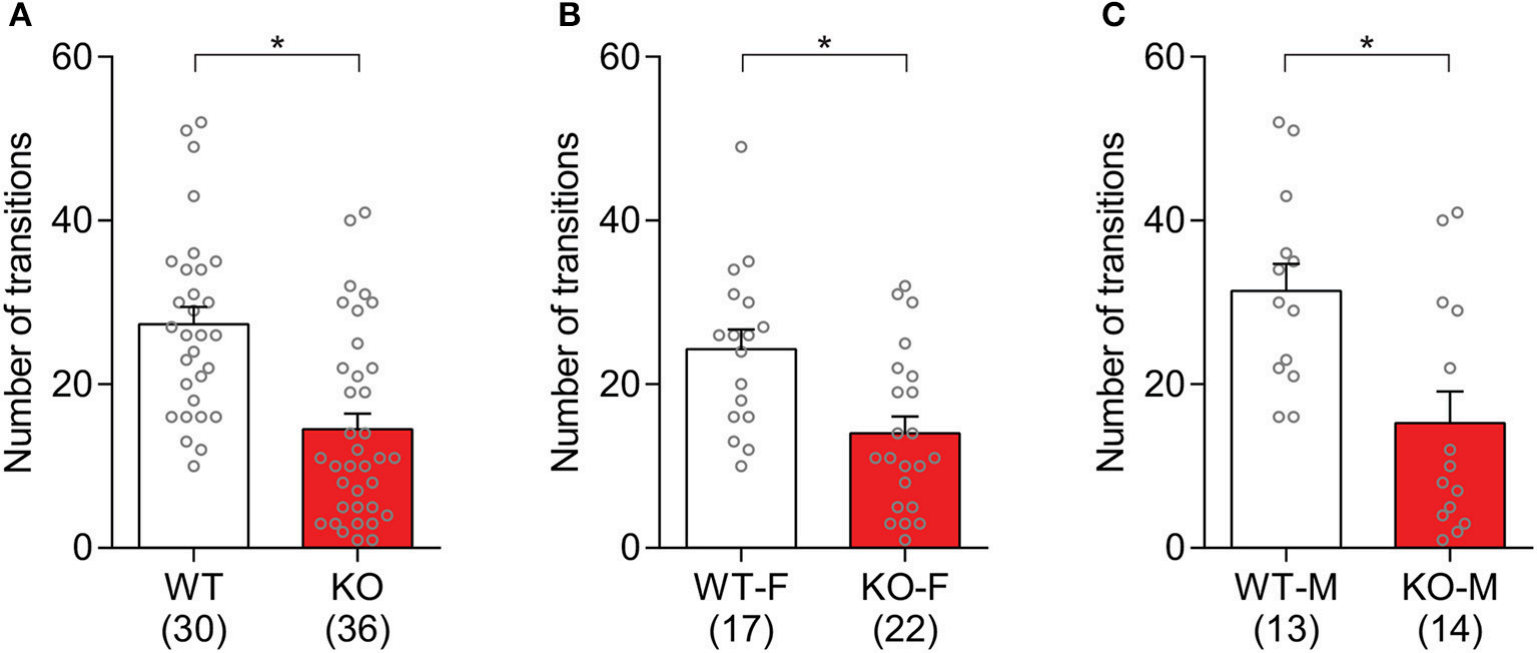
*Slit3*-KO mice showed anxiety-like behaviors in the light/dark box test. **(A)** KO mice made fewer transitions to the light chamber during the 10-min test than WT controls. *p < 0.001, *t*-test. **(B)** Number of transitions to the light compartment in female mice. *p = 0.002, *t*-test. **(C)** Number of transitions to the light compartment in male mice. *p = 0.004, t-test. The data in the bar chart are presented as mean ± SEM. Sample size is indicated in parentheses. F, female; M, male.

The observed anxiety-like behaviors in *Slit3*-KO mice were further assessed based on the number of transitions that animals made between light and dark compartments. The *Slit3*-KO mice displayed increased anxiety-like behaviors as they made significantly fewer transitions between both chambers than WT mice [*t*_(64)_ = 4.57, *p* < 0.001, *t*-test; Figure 7A]. When each sex group was analyzed separately, both male and female KO mice made significantly fewer transitions to the light compartment compared to the relative controls [Female: *t*_(37)_ = 3.26, *p* = 0.002; Male: *t*_(25)_ = 3.16, *p* = 0.004; *t*-test; Figures 7B,C].

### 2.4. Normal object recognition memory and no-depression-like behaviors in *Slit3* KO mice

**Figure 8:**
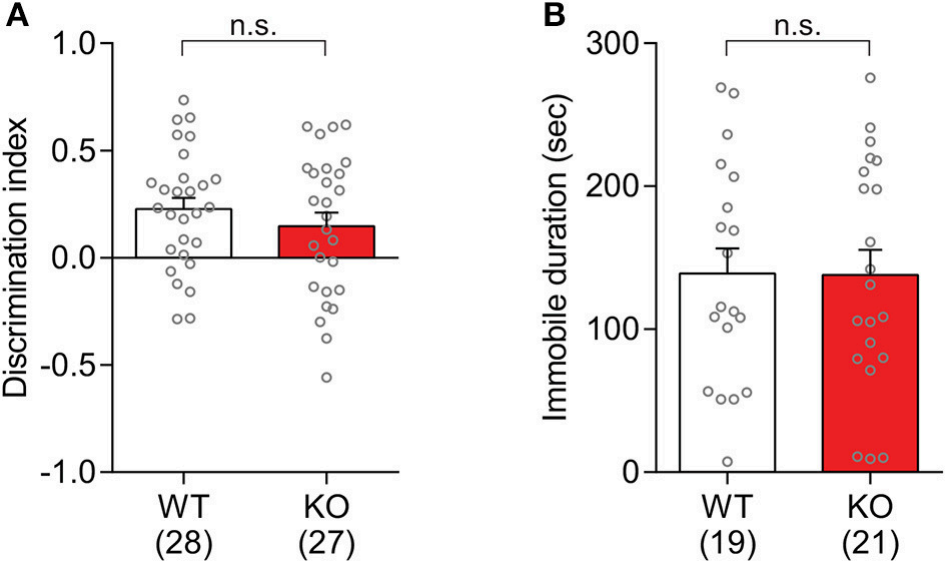
*Slit3*-KO mice were not different from WT mice in the novel object recognition test and tail suspension test. **(A)** There was no significant genotype effect on the novel object discrimination. n.s.: *p* = 0.340, *t*-test. Discrimination index was calculated as Equation (2). **(B)** Immobile duration during the tail suspension test in *Slit3*-KO mice was not significantly different from WT controls. n.s.: *p* = 0.972, *t*-test. The data in the bar chart are presented as mean ± SEM. Sample size is indicated in parentheses.

The hippocampal dependent memory in *Slit3*-KO mice was assessed in the novel object recognition test. *Slit3*-KO mice were able to discriminate between a familiar object that they had explored during the training session 10 min prior to the test session and a novel object. Based on the discrimination index, there was not a significant difference between KO and WT mice [*t*_(53)_ = 0.96, *p* = 0.340, *t*-test; Figure 8A]. The analysis within each sex group did not find any genotype effect [Female: *t*_(23)_ = 0.34, *p* = 0.405; Male: *t*_(28)_ = 1.05, *p* = 0.578; *t*-test].

The depression-like behaviors in *Slit3*-KO mice were assessed in the tail suspension test. The elicited immobility duration in KO mice was not significantly different from that in WT controls [*t*_(38)_ = 0.03, *p* = 0.972, *t*-test; Figure 8B], suggesting that there is no apparent depression-like trait in the *Slit3*-KO mice. The analysis within each sex group did not find any genotype effect [Female: *t*_(28)_ = 0.16, *p* = 0.706; Male: *t*_(8)_ = 0.10, *p* = 0.605; *t*-test].

## 3. Discussion

This study provides novel insight into behavioral phenotypes of the mouse model with the deletion of *Slit3*. Our findings showed that *Slit3*-KO mice exhibited increased anxiety-like behaviors and altered motor behaviors, but normal social approach and preference behaviors. Phenotyping *Slit3*-KO mice in ASD-associated behaviors deepens the understanding of how *SLIT3* mutations may affect ASD symptoms.

Aberrant motor behaviors are the distinct phenotype in *Slit3*-KO mice supported by hypolocomotion in the open field test and impaired motor coordination in the accelerating rotarod task. Although aberrant motor behavior is not the core behavioral criterion of ASD, the impairment in motor function is a symptom strongly associated with ASD (Jeste, 2011; Abu-Dahab et al., 2013). *Slit3* mRNA is expressed in the motor column of spinal cord and cranial motor nuclei during embryonic and postnatal periods, and in the cerebellum and basal ganglia during postnatal periods of rodents (Yuan et al., 1999; Marillat et al., 2002), which supports the significant role that *Slit3* plays in the development and maintenance of neuronal connectivity for motor functions. In addition, the association of single nucleotide polymorphism of *SLIT3* with Parkinson's disease, a disorder mainly focused on motor functions, further supports the importance of *SLIT3* for the maintenance of motor pathways (Lin et al., 2009). Thus, deletion of *SLIT3* may result in aberrant development/maintenance of motor functions and lead to impaired motor coordination or reduced locomotive behaviors in autism.

A high prevalence of anxiety has been found in individuals with ASD and often impacts the quality of daily life (White et al., 2009; van Steensel et al., 2011; Kerns et al., 2014). Female *Slit3*-KO mice displayed stronger thigmotaxis during the first 10 min of the open field test, which indicates that they were more anxious. The increased anxiety-like state in *Slit3*-KO mice was further supported by anxiety-like behaviors observed in the elevated plus maze test and the light/dark box test. *Slit3* mRNA was also detected in the hippocampus, amygdala, and bed nucleus of the stria terminalis of rats during embryonic and postnatal periods (Marillat et al., 2002). As these brain areas are known to be involved in the regulation of anxiety (Calhoon and Tye, 2015; Tovote et al., 2015), the observed expression pattern implicates that *Slit3* may be critical to the development and regulation of neuronal circuits for anxiety. In addition, our results showed that there were sex differences in some behavioral tests. For example, female *Slit3*-KO mice displayed anxiety-like behaviors in the elevated plus maze test and open field test while male KO mice did not. This suggests that the deletion of *Slit3* may influence animal behaviors in a sex-dependent manner, which is supported by evidence that the expression of *Slit3* is sex biased (Hou et al., 2017) and regulated by the female sex hormone, estrogen (Greaves et al., 2014). Moreover, it was reported that, depending on the types of behavioral tests used, mouse strain and gender have different impacts on anxiety measures (Griebel et al., 2000; Võikar et al., 2001; An et al., 2011), which may explain the results that male *Slit3*-KO mice exhibited increased anxiety-like behaviors only in the light/dark box test but not in the other two tests.

In marble burying tests used as a measure of repetitive behaviors, one of the diagnostic criteria for ASD, *Slit3*-KO mice displayed suppressed marble burying behavior. Notably, several other autism-related models also displayed decreased marble burying behaviors despite their increased repetitive behaviors. For example, *Ephrin-A* KO mice (Wurzman et al., 2015), *Shank3*-KO mice (Kouser et al., 2013), and a mouse model of 15q duplication syndrome (Tamada et al., 2010) showed reduced marble burying behaviors. It is possible that reduced marble burying behaviors in *Slit3*-KO mice may reflect the aberrant motor behaviors, which were observed in both the open field test and rotarod test, rather than measuring an alteration in the tendency toward repetitive behavior itself.

Despite the high level of *Slit3* expression in the hippocampus, deletion of the *Slit3* gene did not change object recognition memory in KO mice. It is possible that the presence of SLIT3 in the hippocampus may be involved in anxiety regulation but not memory, as previous studies show that the dorsal and ventral hippocampus regulate memory and anxiety respectively (Fanselow and Dong, 2010). Deletion of *Slit3* may also influence other hippocampal-related cognitive functions such as spatial memory or long-term recognition memory rather than the object recognition memory tested here. Previous studies have found functional changes in *SLIT3* genes to be associated with depression. For example, duplications of the *SLIT3* locus were observed in major depressive disorder cases (Glessner et al., 2010), and families carrying an alteration in *SLIT3* presented with a history of depression (Cukier et al., 2014). However, the tail-suspension test in *Slit3*-KO mice in our study did not find depression-like behaviors in the mice after loss of *Slit3*.

In summary, *Slit3*-KO mice showed aberrant motor behaviors and anxiety-like behaviors, suggesting that *Slit3* may play an important role in neural circuits for motor function and anxiety regulation. The precise mechanism through which *SLIT3* mutations contribute to autism symptoms needs further investigation.

## 4. Materials and methods

### 4.1. Animals

*Slit3* knockout mice were obtained from Dr. Marc Tessier-Lavigne (Stanford University) and originally generated by the research group of Dr. David Ortniz (Washington University) (Yuan et al., 2003). *Slit3* heterozygous with CD1 and C57BL/6 background were bred to obtain *Slit3*^+/+^ (WT), *Slit3*^+/−^ (Het) and *Slit3*^−/−^ (KO). Two- to four-month old male and female WT and *Slit3*-KO mice were used for behavioral tests. Age and sex matched C57BL/6 were used as social partners in three-chamber social tests. All mice were housed 2–5 per cage in ventilated racks in a temperature- and humidity-controlled animal room on a 12h light/dark cycle with lights on from 07:00 to 19:00 and cared by the AAALAC accredited program of the University of Maryland School of Medicine. Autoclaved rodent chow and water were available ad libitum. This study was carried out in accordance with the recommendations of the Guide for the Care and Use of Laboratory Animals, US National Research Council. The protocol was approved by the Institutional Animal Care and Use Committees at the Hussman Institute for Autism and the University of Maryland School of Medicine.

### 4.2. Behavioral tests

Three cohorts of mice were used for behavioral tests (Table 1). The first cohort was tested in the order of: open field test, three-chamber social test, and novel object recognition test. The second cohort of mice was tested in the order of: elevated plus maze, rotarod test, and marble burying test. The third cohort of mice was tested in the order of: elevated plus maze, light/dark box, and tail suspension test. There was at least a 1-week rest period between each test. Animals were moved to the testing room at least 1 h prior to the test. Animals' activity during behavioral assessment was video-recorded by a NIR Monochrome GigE camera or a Logitech C920 HD Pro Webcam, with EthoVision XT software (Noldus, RRID:SCR_002798) used for further offline analysis. All experiments were conducted during the light cycle. Except the elevated plus maze test and the light/dark box test, all other tests were conducted in a room with light at around 80 lux. All arenas were cleaned with 70% ethanol between test sessions.

**Table 1:** Behavioral tests and the number of animals in the three cohorts.

**Cohorts**	**Sex**	**WT**	**KO**	**Behavioral tests**
1st	M	16	18	Open field test^a^, three-chamber social test^b^, and novel object recognition test^c^
	F	17	11	
2nd	M	7	15	Elevated plus maze^d^, rotarod test, and marble burying test^c^
	F	15	12	
3rd	M	13	14	Elevated plus maze^e^, light/dark box, and tail suspension test^c^
	F	17	22	

a *1 KO-F mouse was removed as it was completely immobile during the entire test session*.

b *Data from 8 WT-M, 8 WT-F, 10 KO-M, and 2 KO-F in the social tests were excluded due to a different strain of mice used as social partners*.

c *Some mice were not used for the third test in each cohort when the group size was enough for that test*.

d *1 WT-M, 1 KO-M, and 1 KO-F mice were excluded due to the unexpected noise disturbance or the detection difficulty because of their white fur*.

e *EPM tests were only scored in 7 WT-F, 6 WT-M, and 7 KO-F mice. The rest of animals were still exposed to the EPM for 10 min so that all animals have the same testing experience for the other two tests*.

#### 4.2.1. Three-chamber social test

A slightly modified three-chamber social test was conducted as previously described (Moy et al., 2004; Mei et al., 2016; Park et al., 2016). The test animal was placed in the middle chamber of the three-chamber apparatus (40 × 60 × 20 cm), with left and right chambers closed for 30 s, and then was allowed to explore all three chambers through small openings (10 × 5 cm) with an inverted steel wire pencil cup (10 cm diameter and height) placed in each side chamber for 10 min. At the end of the exploring session, the animal was gently guided back to the middle chamber. During the second session, one novel C57BL/6 mouse was placed under one of the cups and an object was placed under the other, and then the test mouse was allowed to explore all three chambers for 10 min. After that, the test mouse was guided back to the middle chamber. In the third session, the object was replaced by another novel C57BL/6 mouse and then the test mouse was allowed to explore all three chambers again for 10 min. The duration that the animals spent in each zone during each session was scored using the nose point tracking in EthoVision XT software (Noldus). The interaction zone was defined as a circle area 1cm around the pencil cup. The preference index was calculated as:

(1) $$\begin{array}{r} \frac{{\text{D}}_{\text{novel}}}{{\text{D}}_{\text{total}}} \end{array}$$

where D_novel_ is the duration exploring the novel mouse; D_total_ is the total duration exploring the object (or familiar mouse) and the novel mouse.

#### 4.2.2. Marble burying task

Repetitive marble burying behavior was tested as described by Deacon (2006) and Silverman et al. (2010). Twelve glass marbles were evenly placed in a 3 × 4 arrangement on top of 5 cm-deep bedding in standard mouse cages with filter top lids. Each mouse was placed in a cage and allowed to explore for 30 min. The number of marbles buried by the mouse was counted manually. If the marble was buried more than 2/3 deep, it was counted as buried.

#### 4.2.3. Open field test

Animals were allowed to explore a novel open field apparatus (40 X 40 cm) for 30 min to test their locomotive behaviors. In addition, exploration in the center area (20 X 20 cm) during the first 10 min of the test was measured to examine anxiety-like traits, as mice display anxiety-like behavior most often in the beginning of the open field test (first 5–10 min) (Choleris et al., 2001). Distance traveled and time spent in the center area were monitored and analyzed using EthoVision XT software (Noldus).

#### 4.2.4. Elevated plus maze test

The test was conducted under very dim yellow light at around 8 lux. Facing the closed arm in the elevated plus maze apparatus, animals were placed in the center of the elevated plus maze (30 × 5 cm, 15 cm walls on closed arms, 50 cm above the floor) and allowed to explore for 10 min. The duration of time that the mice spent in each arm (animals completely entered the arm) was scored using EthoVision XT software (Noldus). The percentage of time in open arm was calculated as (duration in open arm) / (total duration).

#### 4.2.5. Light/dark box test

The light/dark box test apparatus consists of two parts. The brightness of the light side (40 × 20 cm) was about 600 lux and the dark side (40 × 20 cm) was less than 2 lux. Mice were placed in the center of the light side of the apparatus with an opening (10 × 5 cm) to the dark side. Animals were allowed to explore for 10 min. The number of transitions between chambers was scored using EthoVision XT software (Noldus), and confirmed by manual counting in random samples.

#### 4.2.6. Rotarod test

Animals' motor learning was assessed using the rotarod test (Yang et al., 2012). Animals were tested on the accelerating rotarod (Panlab, Harvard apparatus) over 2 days. Mice were habituated on the stationary rod for 60 s on the first day of testing. During each testing session, mice were placed on the rotating rod (4 rpm) heading opposite to the rotating direction and received accelerating rotarod tasks (4–40 rpm over 5 min). The testing sessions were repeated for three trials a day for 2 consecutive days, with a 1-h inter-trial interval. If the animal fell off the rod within 10 s, it was gently placed back onto the rod. If the animal turned 180 degrees on the rod, it was gently guided back to the original direction. The time delay between the test start and the animals' falling off the rod or making a complete revolution on the rod was recorded as the latency to fall.

#### 4.2.7. Novel object recognition test

Novel object recognition tests were performed as described previously (Cohen and Stackman, 2015). Mice were habituated in the open field box (40 × 40 cm) for 10 min on 3 consecutive days. On the testing day, the mice were allowed to explore two identical objects located in each corner 5 cm from the wall for 10 min. The mice were then placed in an empty cage. After 10 min, the mice were placed back in the box where one of the original objects had been replaced by a novel object and the mice were allowed to explore for 5 min. The duration of time the animals spent in the 2 cm space around the object was scored using EthoVision XT software (Noldus). Discrimination index was calculated as:

(2) $$\begin{array}{r} \frac{{\text{D}}_{\text{novel}}-{\text{D}}_{\text{familiar}}}{{\text{D}}_{\text{novel}}+{\text{D}}_{\text{familiar}}} \end{array}$$

where D_novel_ and D_familiar_ are the duration exploring the novel object and the duration exploring the familiar object, respectively.

#### 4.2.8. Tail suspension test

Depression-like behaviors were tested using the tail suspension test as described previously (Steru et al., 1985; de Rezende et al., 2014). Mice were hung by the tip of the tail taped to the metal rod for 6 min. Immobility time was measured manually.

### 4.3. Statistical analysis

All data were analyzed with Graphpad Prism 6 software (Graphpad Prism, RRID:SCR_002798). All data sets were tested for normality using the D'Agostino-Pearson omnibus normality test. Two-tailed unpaired t-tests, paired t-test, or two-way ANOVA with repeated measures in one factor (rmANOVA) were used for the data with normal distribution. Mann–Whitney analysis was used when the data were not normally distributed. A value of *p* < 0.05 was considered statistically significant.

## Data availability statement

The raw data supporting the conclusions of this manuscript will be made available by the authors to any qualified researcher upon reasonable request.

## Author contributions

CP and SH initiated the project. CP provided the mice. SP collected and analyzed the behavioral assessment data. SP and SH designed the experiment and wrote the manuscript with the help of CP.

### Conflict of interest statement

The authors declare that the research was conducted in the absence of any commercial or financial relationships that could be construed as a potential conflict of interest.
